# Children Base Their Investment on Calculated Pay-Off

**DOI:** 10.1371/journal.pone.0033239

**Published:** 2012-03-08

**Authors:** Sophie Steelandt, Valérie Dufour, Marie-Hélène Broihanne, Bernard Thierry

**Affiliations:** 1 Centre National de la Recherche Scientifique, Département Ecologie, Physiologie et Ethologie, Strasbourg, France; 2 Université de Strasbourg, Institut Pluridisciplinaire Hubert Curien, Strasbourg, France; 3 Université de Strasbourg, Laboratoire de Recherche en Gestion et Économie, EM Strasbourg Business School, Strasbourg, France; CNR, Italy

## Abstract

To investigate the rise of economic abilities during development we studied children aged between 3 and 10 in an exchange situation requiring them to calculate their investment based on different offers. One experimenter gave back a reward twice the amount given by the children, and a second always gave back the same quantity regardless of the amount received. To maximize pay-offs children had to invest a maximal amount with the first, and a minimal amount with the second. About one third of the 5-year-olds and most 7- and 10-year-olds were able to adjust their investment according to the partner, while all 3-year-olds failed. Such performances should be related to the rise of cognitive and social skills after 4 years.

## Introduction

How individuals make choices in the context of interactions with other people is a major topic within economics, and thinking from this discipline has strongly influenced research on decision-making. Trading with multiple partners following different exchange rules is commonplace in the dense social exchange networks developed within human societies. When facing partners offering different expected pay-offs, investors are expected to optimize their satisfaction by adjusting their decision to the most rational choice [1]. Despite the increasing interest of economists in how adults and adolescents decide to invest according to the behavior of other persons [2], we still do not know how this ability appears and develops in an individual. No study to date has investigated the competence of children to calculate investment based on the offers made by partners.

Trust is known to represent a “social lubricant” in the economic world [3], and most models of economic interactions have demonstrated that decision-making is influenced by social preferences such as trust and reciprocity [4]–[7]. The experimental trust game, originally known as the investment game [8], has been used in numerous studies to model the economic behavior of people when trading. In this test, a player typically decides what proportion of an initial monetary endowment will be given to an anonymous player. This amount is then tripled and the recipient decides how much of the tripled amount will be kept and how much will be returned to the first player. Experimental results provide evidence that decision-making is affected by cultural origins [9]–[11], and individual factors such as gender or age [12]–[17]. For instance, men from western societies invest the most [18], [19], and therefore appear to be the most trusting [20], [21] and the most confident in their investment decisions [22], [23]. Adult subjects were also more confident than teenagers [17]. Several studies have shown that reciprocity, trust and fairness affect the decisions of children [24]–[26], but the influence of age on investment decisions in an exchange situation remains little documented (see [27]).

In every transaction, partners not only decide how to trade with one another, but also choose with whom they trade. People may consider alternative, better partners if the current partners do not meet the expected cooperative conditions. In the context of public good provision games, for instance, adults usually adjust their investment following observation of contributions made by partners [28], [29]. In the investment game, interacting several times with the same partner can create a context in which individuals develop trust in previously unknown persons. To explain how trust relationships can evolve over time, researchers have commonly used repeated games in which participants interact several times with the same partner [30]. In studies where subjects can choose their partner, results show that people prefer partners who have already provided them with some form of benefit, and the choice made is based on the past result of their interaction [2], [31], [32]. In contrast, imposing exchange partners on individuals may decrease the level of trust or result in more time being required before participants trust each other [33]. As trusting behavior evolves with age, older children may accept to trade with unknown partners more easily than younger ones would, or they only may decide to take part in the transaction once they have estimated whether the partners were trustworthy.

When confronted by informants who differ by their level of reliability, children consistently prefer the one having given the more accurate information in the past [34]–[37]. Interestingly, an increasing number of works examine economic skills and the influence of partner's reliability in non-human primates [38]–[41]. One study in monkeys showed that a single individual out of twenty-one was able to adapt his investment according to the profitability of two different human partners [41]. It appears that taking the quality of partners into account when trading requires complex cognitive skills. In humans, and especially in children, it is likely that this competence develops in relation with the development of the cognitive abilities needed in any transaction: giving to an unfamiliar person, judging the partner's reliability by reasoning about their mental state, and estimating the value of goods [1], [42].

Studies have shown that children can spontaneously give objects before the age of one [43]–[45]. By 14–18 months of age, they readily interact with unfamiliar people [45] and take part in exchanges with unknown experimenters [46], [47]. Progressive development is also seen in attribution of mental states. At the beginning of the second year of life, children can share goals and read other people' intentions, i.e., the plan of action needed to reach goals [45], [48]–[50]. From 4–5 years of age, children start to understand the beliefs and thoughts of others, which may help them to recognize untrustworthy and dishonest partners [51]–[54]. Regarding numerical skills, children under the age of two can make value judgments by recognizing small discrete quantities [55]–[58], and larger numerosities, albeit in an imprecise way [59]–[63]. At around the age of 2, they are able to count to about six and detect a violation of counting [64]–[67], but children cannot master the same counting principles as adults before six years of age, i.e. the sequence of number words, the one-one correspondence between objects and words, and the cardinal principle [68], [69]. By the age of 5 or 6, they solve verbal calculation problems requiring arithmetic skills [70]–[72], although younger children can already predict the outcomes of simple additions and subtractions [73]–[76].

We aimed to identify the developmental stage at which children adjust their investment in the context of an economic transaction. We tested children between the ages of 3 and 10 in an exchange task requiring them to calculate the amount of food items they gave initially, in order to maximize the food amount to be returned by two different experimenters. The experimental procedure was similar to that used with monkeys [41]. One experimenter gave back a reward twice the amount of the child's initial investment, whereas the other always returned the same amount, whatever the child's initial investment. To maximize pay-offs, children had to respond in different ways to each experimenter, offering a maximal amount to the first one, and a minimal amount to the second.

## Results

### Returned Items

**Figure 1:**
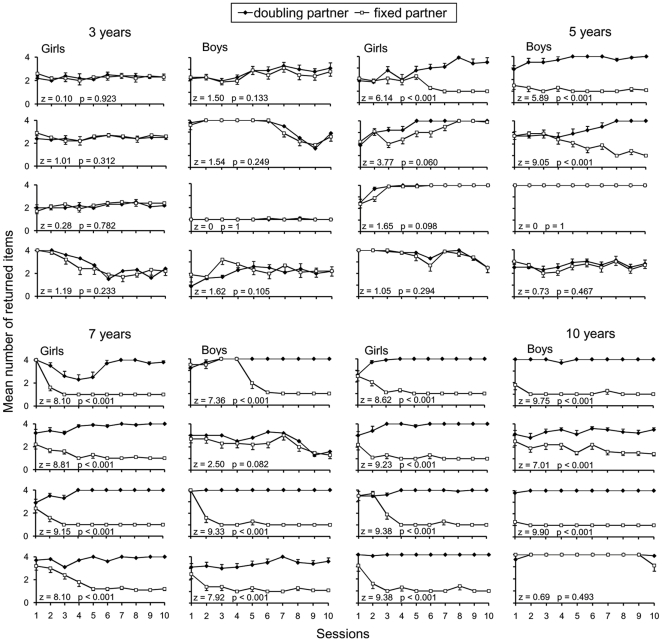
Number of sweets returned by children (n = 8 subjects per age group). No 3-year-old children successfully adapted their strategy according to the quality of partners. At the age of 5, three subjects adapted their strategy according to the quality of partners. Seven subjects successfully adapted their strategy according to the quality of partners at the age of 7 and 10 respectively (Wilcoxon tests, n = 10). Each plot represents the mean number of sweets returned in one session of ten trials, along with standard errors.

The mean number of returned items varied according to individuals (F_24,575_ = 5.5, *p*<0.001; Figure 1), age and partner (3 years: mean number of sweets ± sd = 2.35±0.09, 5 years: m = 3.06±0.07, 7 years: m = 2.58±0.08, 10 years: m = 2.75±0.1, F_1,575_ = 9.4, *p*<0.001; doubling partner: m = 3.29±0.08; fixed partner = 2.08±0.09, F_1,575_ = 58.2, *p*<0.001). Given the interaction between the individuals and partners (F_24,575_ = 2.7, *p*<0.001), we compared the performances of each child according to the quality of partners.

Comparing the performances of 3-year-old children according to the quality of partners did not yield significant differences (Figure 1). Children seldom returned all initial items, often keeping at least one sweet (90.3% of returns).

Among 5-year-old children, five failed to adapt the amount of sweets given according to the quality of their partner (Figure 1). By contrast, three children showed significantly different behavior with each experimenter. One of them adapted his strategy from the first set of sessions; he quickly learned to give back a minimal number of sweets (one) to the fixed partner, and a maximal number (four) to the doubling partner. The other two children modified the amount of returned sweets in the course of experiments, learning to give one sweet to the first partner and four to the other from the 8^th^ set of sessions onwards (Figure 1).

Among 7-year-old children, one boy did not display significant differences in his behavior according to partner's quality; he returned some sweets to both of them (Figure 1). All the other children were able to adjust their behavior according to the quality of partners, most of them learning in the first half of the study to give back a minimal amount to the fixed partner, and a maximal amount to the doubling one (Figure 1).

Among 10-year-old children, one failed to discriminate between experimenters; he repeatedly gave back around four sweets to both partners. All the others adapted their return from the first sets of sessions by giving back a minimal amount of sweets to the fixed partner, and a maximal amount to the doubling one (Figure 1).

### Net Incomes

By experimental design, a child's net income should differ according to the quality of partners. Only one boy of 5 years old did not experience a significant difference of total income between experimenters. For all the other children, income was higher with the fixed than with the doubling partner (Table 1). The difference between the numbers of sweets gained from each partner varied from 14 to 598 sweets (Table 1). It is worth noting that by the age of 5, children regularly counted the net income received during the exchange.

**Table 1:** Children's net income according to the quality of partners and difference between the numbers of sweets received from each partner.

Subjects	Sex	Net incomes		Difference between the number of sweets received from each partner	P-value (Wilcoxon test, N = 10)
		(mean number ± SD)			
		Doubling partner	Fixed partner		
**Three years**					
Ste	Girl	6.3±0.08	9.6±0.11	327	<0.001
Aud	Girl	6.4±0.07	9.3±0.10	286	<0.001
Mil	Girl	6.2±0.08	9.6±0.13	343	<0.001
Cam	Girl	6.8±0.14	9.0±0.18	224	<0.001
Lou	Boy	6.7±0.10	9.5±0.10	285	<0.001
Sim	Boy	7.4±0.10	8.6±0.14	114	<0.001
Matt	Boy	5.0±0.01	10.9±0.01	598	<0.001
Math	Boy	6.0±0.13	9.4±0.17	334	<0.001
**Five years**					
Chl	Girl	6.8±0.12	10.1±0.15	330	<0.001
Gla	Girl	7.5±0.09	9.0±0.12	143	<0.001
Mas	Girl	7.8±0.06	8.3±0.07	49	<0.001
Mar	Girl	7.7±0.09	8.4±0.10	72	<0.001
Mil	Boy	7.7±0.07	10.8±0.09	306	<0.001
Lea	Boy	7.2±0.08	10.0±0.12	277	<0.001
Lou	Boy	8.0±0	8.0±0	0	1
Ami	Boy	6.7±0.10	9.4±0.10	275	<0.001
**Seven years**					
Ami	Girl	7.4±0.11	10.6±0.10	323	<0.001
Fat	Girl	7.7±0.06	10.7±0.07	299	<0.001
Ana	Girl	7.8±0.06	10.8±0.07	303	<0.001
Ass	Girl	7.8±0.10	10.3±0.11	247	<0.001
Ben	Boy	7.9±0.04	9.8±0.14	191	<0.001
Leo	Boy	6.6±0.12	9.8±0.12	315	<0.001
Art	Boy	8.0±0	10.6±0.10	262	<0.001
Leon	Boy	7.0±0.13	8.7±0.39	172	<0.001
**Ten years**					
Ali	Girl	7.8±0.06	10.7±0.07	289	<0.001
Aga	Girl	7.8±0.06	10.8±0.07	299	<0.001
Yae	Girl	7.9±0.06	10.6±0.14	246	<0.001
Mou	Girl	8.0±0	10.6±0.11	263	<0.001
Flor	Boy	8.0±0	10.9±0.05	292	<0.001
Yan	Boy	7.3±0.08	10.2±0.10	286	<0.001
Tho	Boy	8.0±0	11.0±0.03	299	<0.001
Adi	Boy	7.9±0.03	8.1±0.05	14	<0.05

Because some children were from the same school, we could not control potential communication between them about experiments, especially in older children. When looking at the proportion of successful children tested at school versus those tested at home who did not know each other, we found similar proportions in decision patterns, both in 7-year-old children (83% of success at school vs. 100% of success at home), and 10-year-old children (80% vs. 100%).

## Discussion

No 3-year-olds were able to adjust their behavior according to the quality of partners. About one third of the 5-year-old children, and almost all children aged 7 and 10 succeeded in optimizing pay-offs by following different decision rules according to experimenters. The fact that the performances of the children tested at school were not better than those of subjects tested separately at home casts doubt on any possibility of information transmission between children belonging to the same school. It should be added that successful children did not adjust their investment according to the quality of partners from the first trial of a session; they learned to maximize their pay-off after several trials of the first or following sessions. Our results confirm that the ability to calculate investment based on partners' offers develops between 4 and 7 years of age.

It is unlikely that failures observed in 3-year-old children were due to their inability to differentiate between the food amounts returned by experimenters, since children are able to differentiate between discrete quantities from their first year of life [59]–[62], [73], [77]. In the present study, children sometimes returned a different number of sweets to experimenters, thus getting an opportunity to learn that partners did not respond in the same way. Despite having experienced a difference in net income of about three sweets between partners, younger children did not adjust their return according to experimenters' qualities.

In terms of calculation skills, children aged 3 to 4 are able to resolve basic subtractions that involve small number sets [70]–[72], [78]–[80]. Similar abilities are observed in monkeys [81]. Here, children aged 3 are comparable to most macaques and capuchin monkeys tested in the same task [41] as they could remove part of the items before investing, but failed to adapt their return to partners. A single monkey out of twenty one was able to adapt his strategy to both partners, which sets the performances of monkeys somewhere between those of 3- and 5-year-old children. This also suggests that calculation abilities may not be a limiting factor for succeeding in such tasks.

In our experiments, children did not merely have to choose between two options, but also had to draw different decision rules from the contrasting conduct of two partners, which was more demanding. The ability to follow multiple directions or to switch decision rules develops slowly during childhood [82], [83]. When asked to sort objects according to color, 3-year-old children are still unable to inhibit this first representation when required to follow an alternative one based on the shape of objects [84]–[87]. Our study shows that adequate use of opposite decision rules is possible from the age of 5, and is fully mastered from the age of 7. Interestingly, 10-year-old children needed fewer testing sessions (i.e. less than three sessions) than younger ones (i.e. between one and five sessions) before succeeding. It cannot be excluded that it was enough for subjects to separately adjust to each of the partners they were faced with, without comparing their returns. However, younger children failed in the present task despite the fact that, at this age, they should be able to understand the intentions of each experimenter (see [50], [88]) as a partner requiring a certain amount of food to give rewards.

The development of a theory of mind and arithmetic skills may partly explain increasing performances in children. The success of several 5-year-old children is consistent with the fact that 4- and 5-year-old children understand that others may have thoughts and beliefs different from their own [51]–[54], [89], and this ability can be used to detect the reliability of a partner. With regard to arithmetic, it is known that the first years of schooling markedly affect the cognitive skills of children regarding language, literacy and numeracy [90]. Although it appears sufficient to recognize magnitude – even in an imprecise way – for children to adjust their return to partners, the task may ask for a more demanding ability when it comes to understanding the relation function between investing and the return of each partner (when children have to discriminate between ratio differences). School-related changes in counting and arithmetic abilities may lead children to be more efficient when calculating investment. We did not observe children using counting to remove items before exchanging, but most subjects over the age of 5 spontaneously counted the total number of received sweets at each trial. While Jordan, Huttenlocher and Levine (1994) [91] found that counting objects may not be necessary to solve non-verbal tasks, it could help children to differentiate the net incomes received according to the quality of partner and could improve decision-making. A marked increase of performance in arithmetic problem solving is reported from the age of 5 or 6 onwards [68], [71], [72]. Given this long age-related development, it may not be surprising that children only solved the task from the age of 5 onwards. Thus, counting and understanding that people can think differently may improve performance on this task.

Social factors could also have affected the performances of children, and may explain better results found in 5-year-olds. In particular, trusting behavior is commonly believed to guide the choices of economic agents in investment situations [8], [92]. Repeated experiences can establish a trust relationship between exchange partners [30] and this leads children to invest their attention towards how to gain more from the task rather than concentrating on the unknown partner. With increasing age, children could also prioritize the potential to gain more, even if they did not trust experimenters. On the other hand, it is possible that the capacity of younger children to focus on the task was impaired by failure to overcome their wariness of partners who were unknown and potentially untrustworthy. A recent study revealed that 3-year-olds evaluate trustworthiness of partners based on the inaccuracy of information, whereas 4-year-olds rely both on accuracy and inaccuracy [93]. As partners were always trustworthy in our study, systematically giving children an accurate reward, it is quite possible that younger children may have experienced difficulty distinguishing between them. The number of sweets that they kept could also reflect their hesitation to put their trust in experimenters. Contrary to older children, 3-year-olds kept at least one sweet in almost all the trials, thus showing a preference to avoid losing their initial savings rather than acquire gains. Such loss aversion relates to the endowment effect, a cognitive bias commonly found in economics; it leads people to attribute a higher value to objects they own than to objects that they do not possess. A number of experimental studies have demonstrated that adult investors may behave in a way that may not be rational [42], [94]–[96]. Our results showed that children as young as 3 years old also violate the predictions of optimal decision-making models.

Although older children understood how to maximize their benefits, they did not appear perfectly rational since they did not follow an optimal rule on every trial. They often supported their decision verbally by asserting that they preferred to win less for a specific trial, or to set their sights on the contents of a cup that did not contain the maximal amount of sweets. It should be emphasized that children were rewarded regardless of the number of sweets invested; no exchange also rewarded them with the four sweets that they kept. The lack of negative reinforcement for giving one quantity or another can explain that successful children were not always optimizing investors. Such choices probably reveal the importance of play or exploration in their performances.

The present results show that children between the ages of 4 and 10 are in the process of learning how to behave in economic situations. Both cognitive and social factors are likely to be involved in their ability to calculate their investment according to the offers made by partners, and biases appear to influence their decision-making. More research will be necessary to confirm the present results in a larger sample of subjects, and further investigate the relationships between cognitive development and improving economic skills. In particular, it would be worthwhile to study children in an experimental situation that does not involve a social component, for instance, by testing them using two automated dispensers instead of experimenters.

## Methods

### Ethics Statement

The project was approved by both the Education Department of the Bas-Rhin (reference DIVEL1/09-670/IJ) and the district inspector for education. Parents were given a letter describing the general purpose of the study and written parental consent was required for children to participate in the tests. Participation was on an unpaid, voluntary basis, but children kept the sweets that they won during the sessions.

### Participants

We studied 32 children (16 males and 16 females) aged 3 to 10. This sample was divided into four age groups of eight children: 3-year-old (mean age ± SEM = 41.4±4.0 months), 5-year-old (m = 66.5±0.9), 7-year-old (m = 88.0±3.6), and 10-year-old (m = 125.6±3.4). The sex ratio was balanced for representativeness; we tested equal numbers of girls and boys in each of the four age groups, i.e. four girls and four boys. An additional child was excluded from tests because he did not pay any attention to the experiments.

Participants were European, from middle-class backgrounds, with French as their first language. A majority of children belonged to the Robertsau preschool and elementary school in Strasbourg, France. We tested seven children separately (two 3-year-olds, two 7-year-olds and three 10-year-olds) at their home, i.e., outside the frame of the school.

### Experimental Design

We studied children in two conditions involving different experimenters. In the first case, the experimenter was a doubling partner, meaning that she always returned twice the number of rewards given by the subjects; potential rewards were presented in four cups containing either two, four, six or eight rewards. In the second condition, the experimenter was a fixed partner, meaning that she always returned eight rewards, regardless of the number of rewards given by subjects (one to four); potential rewards were presented in four cups, each containing eight sweets.

Each child took part in two sessions, one session of 10 trials each, with each of the experimenters. A session was composed of 10 trials separated by pauses of 5 sec. Children were given 5 min between the two sessions to fully understand the different conduct of the two experimenters. The net income, i.e. the amount of the rewards kept by the children plus those received, could vary within any one session from 24 to 48 sweets with the doubling partner, and from 24 to 66 sweets with the fixed partner (Table 2). We counterbalanced the role of experimenters, i.e. within each age-and-sex group, one experimenter was the doubling partner with two children, and was the fixed partner with the other two. We also alternated the intervention order of partners from one set to another. To help children learn to distinguish between both conditions, we associated them with different cues. We divided the table into two parts, each devoted to a different set of four cups, with a different color for each condition.

**Table 2:** Number of rewards acquired from both experimenters and children's net income according to the number of items returned by children.

	Doubling partner		Fixed partner	
Number of sweets returned	Reward	Net income	Reward	Net income
0	0	4	0	4
1	2	5	8	11
2	4	6	8	10
3	6	7	8	9
4	8	8	8	8

Within any one session, the subjects' net income is the amount of items kept by the child plus those received after return. Subjects maximize their gain by giving more (4 sweets, net income 8) to the doubling partner, and less to the fixed partner (1 sweet, net income 11).

### Test Procedure

We recorded whether subjects had siblings or twins. We videotaped testing sessions whenever written consent was obtained. Children were tested individually in a quiet room (4 m×3 m) adjoining their classroom. The child was led to the testing room and introduced to the two experimenters. The child then sat on a chair opposite the experimenters at a rectangular table (0.8 m×0.5 m).

Before testing, the experimenter gave the child the possibility to exchange one reward for two. If they failed, the experimenter repeated the trial once. If the child failed again, the test was stopped. When the child was successful, the experimenter then offered her/him the possibility of exchanging two rewards for four. If s/he failed, we repeated the offer once. All subjects reached this stage, and were considered ready for testing.

The two sessions lasted approximately 15 min. The first trial began when the first experimenter (doubling or fixed partner) placed and filled the four plastic cups with the different rewards. The experimenter then gave the child four sweets by placing them on the surface of the table while saying, “Here are four sweets and here are more sweets”, showing the cups of sweets. After 3 sec, she pointed to the four sweets, held out her hand, palm up, in front of the child and asked, “How many of them do you want to exchange?” (Note that pilot trials run with children aged between 4 and 12 years revealed that the sentence “Do you want to exchange any of them?” implied that the transaction may be risky, leading children either to accept exchanging all the items or to refuse exchanging any of them. We opted for a more precise question that made clear that the children did not have to return all the items).

Every time the child returned one or several sweets, the experimenter thanked them and presented the corresponding cup to the child saying “OK, here are the rewards. Do you want to try again?” before starting another trial. When the child kept or consumed all sweets, the experimenter said “OK, you can eat/keep the sweet(s). Do you want to try again?” After the session, the experimenter said “OK, that was great. Now you're going to play another game with my friend”. The second experimenter (doubling or fixed partner) drew attention to the change of condition by placing and filling four other plastic cups with a different amount of rewards, and then began the second testing session.

### Control of Information Transfer

To avoid any exchange of information between children tested within the same school, the experimenter asked the child not to talk about testing with other children after the first two sessions were completed. It is also important to note that younger children were not verbally mature enough to have elaborate discussions with school friends [97] about how to gain more rewards during experiments.

### Statistical Analysis

We used a one-way repeated-measures ANOVA (Mauchly's test for sphericity = 0.74) to assess the effect of the individuals, age, sex and partner on the mean number of returned items. To test whether subjects responded differently to the fixed and doubling partners, we compared their performances at the individual level using the Wilcoxon matched-pairs test (exact procedure [98]) with SPSS software version 17 (SPSS Inc., Chicago IL, U.S.A.). The significance level was set as 0.05. Values are given as means and standard errors of the means.
